# Limits to lifespan growth

**DOI:** 10.3389/fpubh.2022.1037544

**Published:** 2023-01-06

**Authors:** Marta Gonçalves, Byung Mook Weon

**Affiliations:** Soft Matter Physics Laboratory, School of Advanced Materials Science and Engineering, SKKU Advanced Institute of Nanotechnology (SAINT), Sungkyunkwan University, Suwon, South Korea

**Keywords:** lifespan, survival curve, supercentenarians, mathematical constraints, stretched exponential model

## Abstract

A long-standing human lifespan debate is revival, and the consensus is yet to come on whether the maximum human lifespan is reaching a limit or not. This study discusses how mathematical constraints inherent in survival curves indicate a limit on maximum lifespans, implying that humans would have inevitable limits to lifespan growth.

## 1. Introduction

The existence of the maximum human lifespan is an active debate issue. Recent estimations suggest a limit of the maximum human lifespan of around 125 years (1, 2). In contrast, others disagree with possible limits to lifespan growth. To reconcile this contrast, we must consider additional limiting factors for human lifespan growth.

The survival rate *s*(*x*) is typically a monotonic decrease function of age *x* and a mathematical equivalent of the mortality rate as μ(*x*) = –*dln*(*s*(*x*))/*dx*. The first-successful Gompertz model (3) to describe human survival curves assumes the exponential growth of the mortality rate as μ(*x*) = *a*exp(*bx*) with two parameters *a* and *b* or equivalently ln(μ(*x*)) = ln(*a*)+*bx*, but proves not appropriate to describe the mortality rate at extreme ages (over 100 years). Generally, survival curves can vary by external (ecological or social) conditions under internal (biological) constraints, which makes survival curves plastic, and the ultimate evolution of survival curves becomes rectangular (4). Because of the plasticity and rectangularity of human survival curves, it is essential to find flexible mathematical models more appropriate than the Gompertz model (5). The recent debate on the existence of the maximum human lifespan is due to different interpretations and predictions even for the same demographic data (1, 6–8). There is no consensus on appropriate mathematical constraints of survival curves.

In this study, we consider mathematical constraints inherent in survival curves, featured with plasticity and rectangularity for humans, and discuss the existence of the maximum human lifespan based on a quite flexible mathematical model for human survival curves. This study eventually suggests that humans would have inevitable limits to lifespan growth.

## 2. Materials and methods

The periodic life tables for Sweden females and Japan females between 2010 and 2020 as representative demographic data for humans are taken from the Human Mortality Database (available at https://www.mortality.org/) devoted by Max Planck Institute for Demographic Research (Germany), University of California, Berkeley (USA), and French Institute for Demographic Studies (France). The demographic information on supercentenarians is taken from the International Database on Longevity (IDL) (available at https://www.supercentenarians.org). The mortality database for women was chosen because of their longer lifespans and the usual representation of the highest life expectancy in each country, which is compatible with this study's critical purpose of addressing the limits of lifespan growth (9–11). To understand the limits of human survival, a similar approach can be used to analyze the patterns in male mortality.

**Figure 1:**
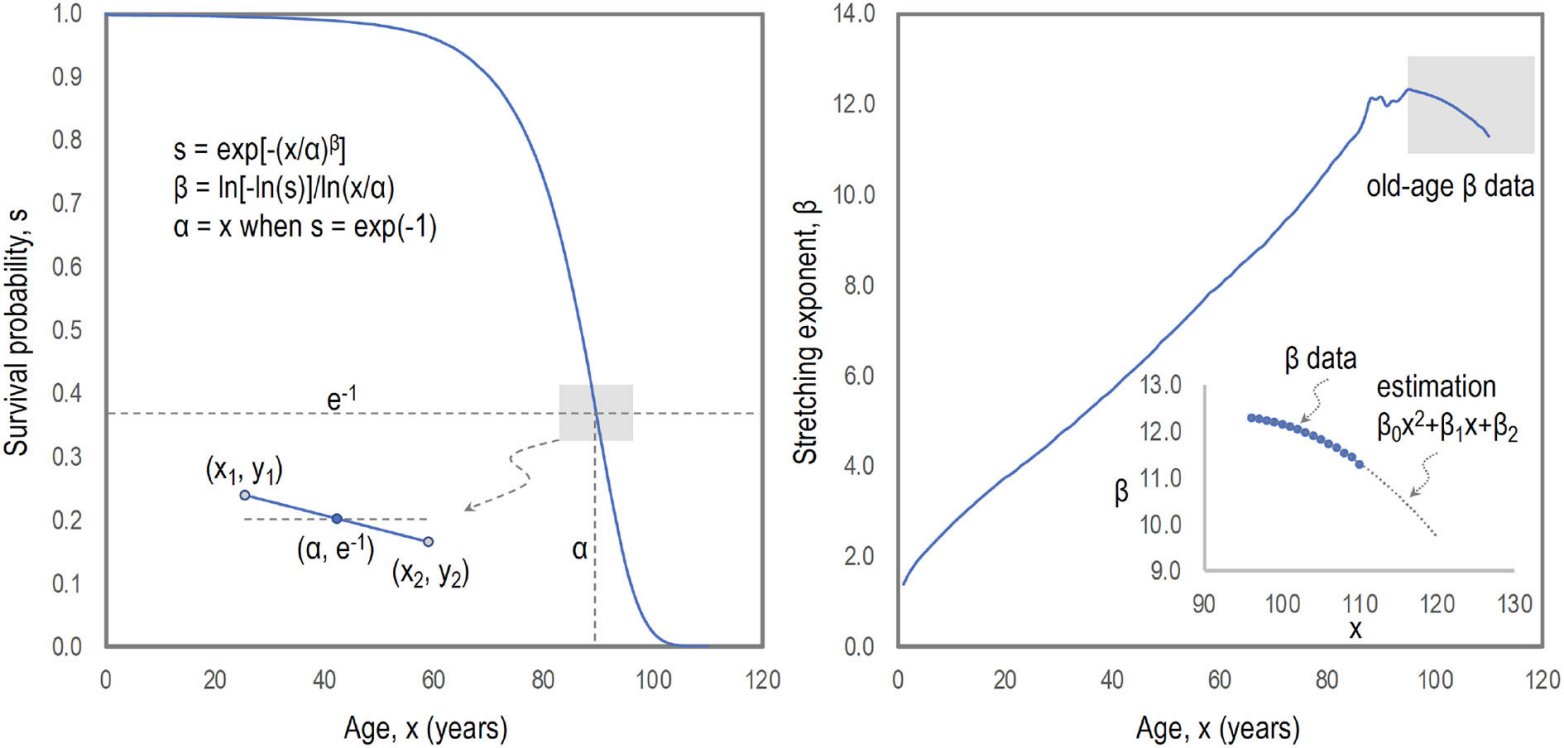
Mathematics of survival curves. Human survival curves can be plastic and tend to be almost rectangular. Such plasticity and rectangularity can be described by adopting the modified stretched exponential model as *s*(*x*) = exp[−(*x*/α)^β(*x*)^] featured with the characteristic age α for *s*(α) = *e*^−1^ (the crossover of *s*(*x*) and *e*^−1^) and the age-dependent stretched exponent β(*x*). The age dependence of the stretched exponent at extreme ages (over 100 years) is described as a quadratic model of β(*x*), which is realistic regarding the plasticity and rectangularity of human survival curves.

Biological survival curves look quite plastic to ecological or social conditions. The recent trends of human survival curves seem to have become plastic and rectangular since the survival strategies have become optimized and deaths occur predominately at higher ages. Such plasticity and rectangularity of human survival curves can be described by adopting the modified stretched exponential model (identical with the extended Weibull model (12)) formulated as *s*(*x*) = exp[−(*x*/α)^β(*x*)^] with the characteristic age α taken at *s*(α) = *e*^−1^ and the age-dependent stretched exponent β(*x*). The age dependence of the stretched exponent differs from the stretched exponential model fixed at constant β (≠1.0) and the simple exponential model at β = 1.0 (13). Practically, the quadratic model for β(*x*) describes the actual survival or mortality curves at extreme ages, as illustrated in Figure 1 for Sweden females (2020).

The rectangularity of human survival curves is simply formulated by β(*x*)≈7/ln(*x*) from the modified stretched exponential model, which corresponds to *s*(*x*)≈1 for *x* < α and *s*(*x*)≈0 for *x*>α (14). The mortality curves at extreme ages (over 100 years) are quite well described as the quadratic models of β(*x*), which are realistic regarding the plasticity and rectangularity of human survival curves (15).

**Figure 2:**
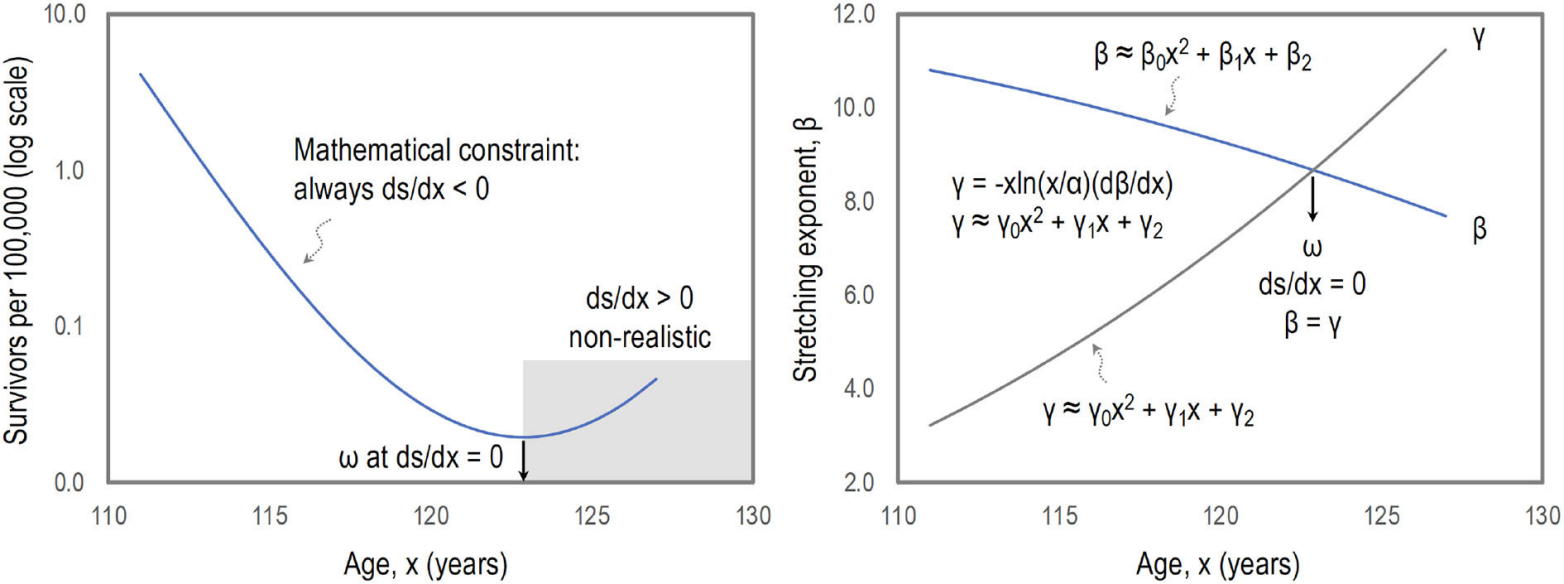
Mathematical constraints of survival curves. The monotonic decrease of survival curves is inherent as *ds*/*dx* < 0, and the increase of survival curves or *ds*/*dx*>0 is non-realistic, providing the maximum mathematical lifespan (ω). Defining ω at *ds*/*dx* = 0, which is equivalent to β(*x*) = γ(*x*) for the modified stretched exponential model, we are able to estimate ω at the crossover of the plausible quadratic models of β(*x*) and γ(*x*).

The monotonic decrease of survival curves is inherent as *ds*/*dx* < 0 and the increase of survival curves or *ds*/*dx*>0 is non-realistic. Therefore, the mathematical feature of survival curves must provide the maximum mathematical lifespan (ω) at *ds*/*dx* = 0. Defining ω at *ds*/*dx* = 0, which is equivalent to β(*x*) = γ(*x*) where γ(*x*) = −*x*ln(*x*/α)(*dβ*/*dx*) for the modified stretched exponential model, we are able to estimate ω at the crossover of the plausible quadratic models of β(*x*) and γ(*x*), as illustrated in Figure 2 for Sweden females (2020).

## 3. Results and discussion

**Figure 3:**
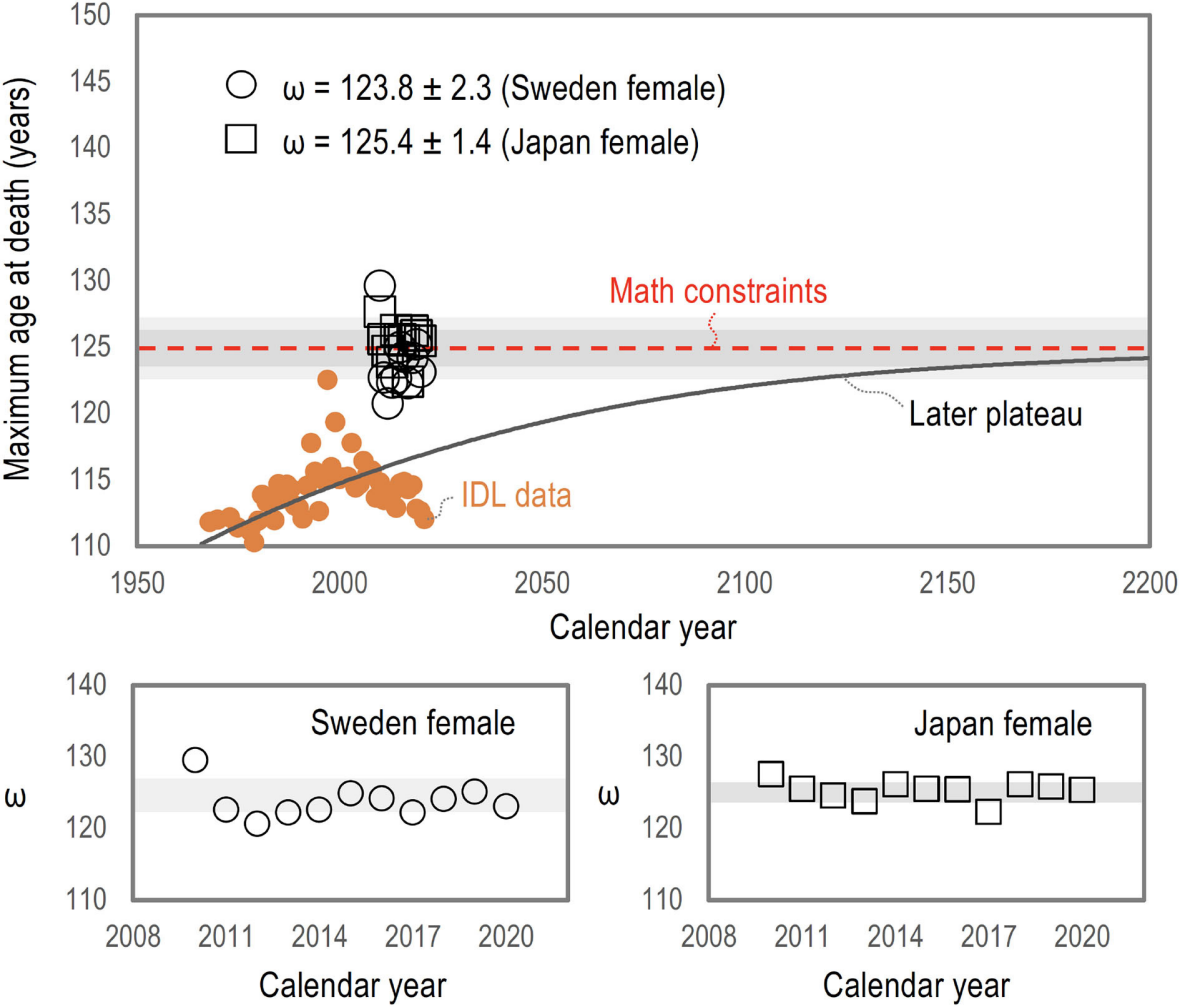
Possible limits to human lifespan growth. The maximum mathematical lifespans (ω) taken from periodic life table data for Sweden females and Japan females between 2010 and 2020 are almost constant as ω = 123.8 ± 2.3 years for Sweden females and ω = 125.4 ± 1.4 years for Japan females (± a standard deviation, which is described by a shaded area). The actual survival curve cannot surpass the maximum mathematical lifespan limit, indicating the existence of mathematical constraints around ~125 years (marked by the dashed red line). This consideration is consistent with the later plateau hypothesis of the maximum age at death (taken from Hughes and Hekimi (16), marked by the solid black line) among three possible expectations of the IDL data (dots) for supercentenarians.

The maximum mathematical lifespans (ω) taken from periodic life table data for Sweden females and Japan females between 2010 and 2020 are almost constant as ω = 123.8 ± 2.3 years for Sweden females and ω = 125.4 ± 1.4 years for Japan females (ω= average ± a standard deviation), as demonstrated in Figure 3. The actual survival curve is invalid over the maximum mathematical lifespan because of *ds*/*dt* < 0, indicating the existence of mathematical constraints around ~125 years (marked by the dashed red line). This consideration is consistent with the later plateau hypothesis of the maximum age at death (marked by the solid black line) among three possible expectations of the IDL data (dots) for supercentenarians (16).

The mathematical constraint presented here gives more malleability when predicting the future maximum human ages of death. Previously, researchers have identified a human lifespan plateau at around 115 years (7, 17), claiming that human life expectancy had an early limit defined by biological barriers and based on the last decades of mortality data. However, the later plateau of the maximum age at death at around ~125 years, confirmed by the mathematical constraint, is a more reasonable prediction when considering the increment of supercentenarians and their probability of reaching ages past 115 years (1).

Furthermore, the existence of a mathematical constraint, as shown in Figure 3, defined by *ds*/*dx* = 0, narrows down the possibilities of human lifespan growth suggested by several mathematical models, agreeing well with a later plateau of around ~125 years, as indicated by other researchers (1, 16). This approach effectively points toward a more realistic maximum age of death that follows the current trend of supercentenarians and predictions for the next decades. Employing mathematical constraints becomes a handy criterion when assessing the survival rates and lifespan with the several proposed models, helping to find a consensus for the multiple approaches besides biological, evolutionary, and environmental constraints (18).

The human lifespan limit remains one of the oldest research questions that spark heated arguments surrounding the possible answers. The rising number of supercentenarians has questioned the applicability of mathematical models like the Gompertz model. Various researchers may make different predictions because of the availability and reliability of data for older ages. Even for the same datasets, there may be discrepancies, mainly due to mathematical modeling and interpretation. Generally, a consensus regarding reliable databases and appropriate mathematical models will lead to predictions toward a more accurate lifespan limit.

## 4. Conclusion

This study demonstrates that the mathematical constraints inherent in survival curves can predict the maximum human lifespan growth limit. This result implies that humans would reach an inevitable later plateau toward the actual maximum lifespan limits.

## Data availability statement

The original contributions presented in the study are included in the article/supplementary material, further inquiries can be directed to the corresponding author.

## Author contributions

BW conceived the study and collected the data. MG and BW analyzed the data and interpreted the results, wrote, and reviewed the manuscript. All authors contributed to the article and approved the submitted version.
